# Pilot Testing of the Promoting Positive Care Interactions (PPCI) in Assisted Living Study During a Pandemic

**DOI:** 10.1093/geroni/igab046.1203

**Published:** 2021-12-17

**Authors:** Anju Paudel, Elizabeth Galik, Barbara Resnick, Kelly Doran, Marie Boltz, Shijun Zhu

**Affiliations:** 1 Penn State Ross and Carol Nese College of Nursing, University Park, Pennsylvania, United States; 2 University of Maryland School of Nursing, Baltimore, Maryland, United States; 3 Pennsylvania State University, University Park, Pennsylvania, United States; 4 University of Maryland, Baltimore, Maryland, United States

## Abstract

The purpose of this study was to test the feasibility and preliminary efficacy of Promoting Positive Care Interactions (PPCI)—a four step intervention designed to establish positive care interactions between staff and residents with cognitive impairment or dementia in Assisted Living (AL). Initially designed as a traditional on-site intervention, PPCI was later transformed to be conducted remotely through webinar and virtual meetings due to challenges related to onsite engagement in AL during the COVID-19 pandemic. Additionally, the study adopted shorter timeline, a single group pretest-posttest design, and limited recruitment to staff only; 17 care staff were recruited, and data was collected via online surveys and interviews. PPCI was successfully implemented as intended with considerable stakeholder engagement. Findings demonstrated feasibility and promising staff adoption of PPCI. Continued research is needed to optimize the quality of care interactions in AL and evaluate whether online approach to staff training can change staff behavior.

